# RNA-Binding Protein Dnd1 Promotes Breast Cancer Apoptosis by Stabilizing the Bim mRNA in a miR-221 Binding Site

**DOI:** 10.1155/2017/9596152

**Published:** 2017-01-16

**Authors:** Feng Cheng, Ying Pan, Yi-Min Lu, Lei Zhu, Shuzheng Chen

**Affiliations:** ^1^Department of Thyroid-Breast Surgery, Lishui Municipal Central Hospital, Lishui Hospital of Zhejiang University, The 5th Affiliated Hospital of Wenzhou Medical University, Zhejiang 323000, China; ^2^Department of Oncology Surgery, The First Affiliated Hospital, College of Medicine, Zhejiang University, Hangzhou 310003, China

## Abstract

RNA-binding proteins (RBPs) and miRNAs are capable of controlling processes in normal development and cancer. Both of them could determine RNA transcripts fate from synthesis to decay. One such RBP, Dead end (Dnd1), is essential for regulating germ-cell viability and suppresses the germ-cell tumors development, yet how it exerts its functions in breast cancer has remained unresolved. The level of Dnd1 was detected in 21 cancerous tissues paired with neighboring normal tissues by qRT-PCR. We further annotated TCGA (The Cancer Genome Atlas) mRNA expression profiles and found that the expression of Dnd1 and Bim is positively correlated (*p* = 0.04). Patients with higher Dnd1 expression level had longer overall survival (*p* = 0.0014) by KM Plotter tool. Dnd1 knockdown in MCF-7 cells decreased Bim expression levels and inhibited apoptosis. While knockdown of Dnd1 promoted the decay of Bim mRNA 3′UTR, the stability of Bim-5′UTR was not affected. In addition, mutation of miR-221-binding site in Bim-3′UTR canceled the effect of Dnd1 on Bim mRNA. Knockdown of Dnd1 in MCF-7 cells confirmed that Dnd1 antagonized miR-221-inhibitory effects on Bim expression. Overall, our findings indicate that Dnd1 facilitates apoptosis by increasing the expression of Bim via its competitive combining with miR-221 in Bim-3′UTR. The new function of Dnd1 may contribute to a vital role in breast cancer development.

## 1. Introduction

RNA-binding proteins (RBPs) play vital roles in regulating RNA biology through the interplay with RNAs within dynamic ribonucleoproteins [1]. Numerous diseases have been linked to abnormal expression of RBPs, including metabolic disorders [2], germ-cell development [3], muscular atrophies [4], and cancer [5].

Dnd1, an evolutionary conserved RBP, is implicated in mediating germ-cell activity and inhibits the germ-cell tumors formation [6] and regulates the male germ-cell development by serving as an essential partner of NANOS2 [3]. On the other hand, Dnd1 expression is repressed in primary acute myeloid leukemia patients and inhibition of Dnd1 mRNA expression significantly attenuated NB4 differentiation [7]. Moreover, Dnd1 expression change mediated by miR-24 could suppress the expression of cyclin-dependent kinase inhibitor 1B (CDKN1B) and also led to enhanced proliferation and reduced apoptosis in tongue squamous cell carcinoma cells [8]. These findings imply that Dnd1 not only regulates germ-cell development but also has wide-ranging roles in tumor development, like in skin oncogenesis [9]. However, limited information is available on the role of Dnd1 in breast cancer.

3′UTR has various effects on mRNA translation, subcellular localization, and stability through the multiple regulatory elements within them [10]. And 3′UTR could exert tumor-suppressive or tumor-promotive effects via regulating gene expression [11, 12]. In addition, the recent exploration of 3′UTR functions suggests the potential involvement of 3′UTR as a new marker in the preclinical oncology [13]. Therefore, focusing on 3′UTR-mediated effects may provide novel opportunities for 3′UTR-based therapy. Most importantly, Dnd1 has been proved to counteract the function of several miRNAs in human cells by binding to mRNA 3′UTRs and thus inhibiting the association of miRNAs with their target mRNAs [14]. miRNAs are small noncoding single-stranded RNA molecules that regulate gene expression at a posttranscriptional level via binding with the 3′UTR [15]. However, the counteracting roles of Dnd1 with miRNAs in breast cancer development remain unclear.

Based on the above-described link of Dnd1, 3′UTR, and miRNA in tumor development, we investigate their roles in apoptosis of breast cancer. The expression level of Dnd1 was examined in 21 breast cancerous tissues paired with adjacent tissues through qRT-PCR analysis. We further annotated the TCGA microarrays samples by taking into account gene expression levels of breast cancer and analyzed the expression correlation between Dnd1 and other genes. We revealed that low Dnd1 level was a potential marker for breast cancer and that the expression level of Dnd1 was correlated with Bim, which is hall-marker of apoptosis. miR-221 has been proved to promote laryngeal cancer proliferation by suppressing Apaf-1 [16]. Most importantly, miR-221 has been proved to inhibit the apoptosis via targeting Bim or confer a poor prognosis in breast cancer patients [17, 18]. As the specific counteracting between Dnd1 and Bim, it is reasonable to assume that Dnd1 binds to Bim-3′UTR through counteracting miR-221 function in breast cancer.

Here, we first survey the levels of Dnd1 and Bim in clinical breast cancer tissues and microarray, aiming to investigate whether the expression levels of Dnd1 and Bim are correlated with breast cancer development and whether the expression level of Dnd1 is positively correlated with Bim level. In addition, we focus on the effect of Dnd1 on breast cancer apoptosis* in vitro*. Finally, the mechanism of the interaction between Dnd1 and Bim is identified, in order to determine whether Dnd1 competitively bind to the Bim-3′UTR with miR-221, thus promoting breast cancer apoptosis. To the best of our knowledge, the function of Dnd1 validated here has not yet been reported anywhere. Together, we show that the RBP Dnd1 prohibits miRNA-dependent inhibition of Bim expression in breast cancer cells and underscores the tumor-inhibitory roles of the Dnd1 in breast cancer apoptosis, so it may be a possible therapeutic target of breast cancer.

## 2. Materials and Methods

### 2.1. Cell Culture and Patient Samples

Human breast cancer cell line MCF-7 and normal mammary gland epithelial cell line HBL-100 were purchased from the Cobioer Bioscience Co., Ltd., and 67NR, 168FARN, 4TO7, 66cl4, and 4T1 were purchased from the cell bank in Chinese Academy of Sciences of China. All of the above cell lines were maintained in a 37°C, 5% CO2 incubator in DMEM medium (Gibco, Grand Island, NY, USA) supplemented with 10% FBS (fetal bovine serum) (Gibco) and antibiotics with 5% CO_2_ at 37°C under humidified atmosphere. Twenty-one pairs of breast tumors with neighboring mammary normal epithelial tissues were obtained from 21 patients who underwent surgery at the Central Hospital of Lishui City from February 2015 to December 2015 and all the 21 cases had no metastasis. Approval from the Institute Research Ethics Committee was obtained for the use of these clinical materials for research purposes. Paired mRNA profiling data was downloaded from the TCGA data portal (http://cancergenome.nih.gov/) and a total of 351 TCGA breast cancer samples were utilized as a research set. The dataset from the Tumor Breast-EXPO-351-MAS5.0-u133p2 (http://hgserver1.amc.nl/cgi-bin/r2/main.cgi), which includes 351 breast cancer samples, was acquired as a discovery set. The microarray data set was reserved in the Gene Expression Omnibus (GEO) (accession number GSE2109) according to “minimum information about a microarray experiment” (MIAME) guidelines. The R2 platform was used to analyze the microarray (http://r2.amc.nl/).

### 2.2. Plasmid and Reporter Constructs

Reverse transcription PCR (RT-PCR) was used to obtain the human Bim and Dnd1 complementary DNA (cDNA) in MCF-7 cells, followed by amplification of the 3′UTR and 5′UTR of Bim mRNA using a cDNA template and the following the primers: Bim-3′UTR forward: 5′-GAATCCACTTCGCTCCGCGCAGCCGCCTGGT-3′ and reverse 5′-GGATCCTTGGTCTTTTTTTCTTGCGTTTCTC-3′. The PCR products were subcloned into EcoRI- and BamHI-digested pd2EYFP-N1 reporter vector (Clontech, CA, USA): Bim-5′UTR forward: 5′-GAATCCCTGGTCTGCAGTTTGTTGGAGCTCT-3′ and reverse 5′-ACCGGTCAGTTATTTACAGCAGTATTGCACA-3′. The PCR products were subcloned into EcoRI- and AgeI-digested pd2EYFP-N1 reporter vector. And miR-221-mutated binding site in YFP-Bim was inserted by employing overlap extension Polymerase Chain Reaction (PCR). Bim promoter sequence was introduced into the pGL3 vector (Promega) for Bim promoter transcriptional activity assay. The coding sequence of Dnd1 was cloned into the plasmid pcDNA3.1(+), referred to as Dnd1-CDS. Primer sequences were as follows: forward: 5′-AAGCTTATGCAGTCCAAGCGGGATTGTGAGC-3′; reverse: 5′-GAATCCTCACTGTTTAACCATGGTACCTGCC-3′.

### 2.3. Transfection

For the transfection of siRNAs (Santa Cruz), synthetic siRNAs were transfected into cells that reached 40–60% confluence at a final concentration of 50 nM using Lipofectamine 2000 (Invitrogen, Carlsbad, CA) in 6-well plates following the recommending protocols. And a universal negative control siRNA (siRNA NC) was used. For the transfection of plasmids, cells that reached 80% confluence were transfected with 2.5 *μ*g plasmid using Lipofectamine 2000 in 6-well plates following the recommending protocols. 35 nM siRNA or miRNA mimics or anti-miRNAs and 1.6 *μ*g plasmid were used for cotransfection with Lipofectamine 2000.

### 2.4. Real-Time Quantitative PCR (qRT-PCR)

Total RNAs were extracted from cells using the Trizol (Invitrogen, USA) reagent according to the manufacturer's instructions. The first-strand cDNA was synthesized using M-MLV (Promega, USA) following standard protocols. Dnd1, Bim, and GAPDH mRNA expression levels were detected using the specific primers and SYBR Green Master Mix (Biomics Biotechnology Inc., China). The miRNA first-strand cDNA was generated with miRNA reverse transcription kit (Abm, Canada). miRNA qRT-PCR kit and miR-221 primer were purchased from Abm. U6 snRNA was used as an endogenous quantity control for miRNA quantification. And qRT-PCR was performed on an ABI Prism 7500 Sequence Detector (Applied Biosystems, Life Technologies). Melting curve analysis was used routinely to check the specificity of amplification.

### 2.5. Western Blot

Detailed procedure was described elsewhere [19]. The antibodies against Dnd1 (Catalog number ab104792) and *β*-actin (Catalog number ab8226) were purchased from Abcam, the antibodies against Bim (sc-11425), PARP (sc-23461-R), and caspase-3 (sc-65496) were purchased from Santa Cruz. Protein expression levels were quantified by density analysis using Quantity One Software and normalized to *β*-actin.

### 2.6. RIP (RNA Immunoprecipitation) Assays

MCF-7 cells were lysed with 25 mM Tris-HCl buffer (pH 7.5) and 100 U/mL RNase inhibitor (Sigma), and then whole-cell extracts were incubated with protein-A Sepharose beads precleared with anti-Dnd1 antibody or anti-Ago2 antibody or control rabbit IgG for 2 h at 4°C. After washing with NT2 buffer, the beads were incubated with 30 U of RNase-free DNase I in NT2 buffer for 20 min at 37°C, regulation of Bim mRNA turnover by Dnd1 (Invitrogen) in NT2 buffer for 30 min at 37°C and further incubated in NT2 buffer containing 0.2% SDS and 0.5 mg/mL proteinase K for 20 min at 55°C. RNA was extracted with Trizol and mRNA levels were measured by qRT-PCR.

### 2.7. Clinical Data

The KM Plotter tool (http://kmplot.com/analysis/) [20], a meta-analysis-based biomarker assessment tool, was used to compare the survival of breast cancer patients whose Dnd1 and Bim mRNA levels were in the top 1/3 (high) versus the bottom 1/3 (low) groups in publicly available breast cancer gene expression data (Affymetrix (Santa Clara, CA, USA) ProbeID) and selected on the basis of the following parameters: overall survival (OS, 1117 patients), upper versus lower tertile of Dnd1 expression, and including both ER positive (+) and negative (−) breast cancer.

### 2.8. Statistical Analysis

All data are presented as mean plus SD. For three independent experiments, the differences between the groups were analyzed with Student's *t*-test, and ^*∗*^ *p* < 0.05 or less was considered significant.

## 3. Results

### 3.1. Dnd1 Expression Level Is Reduced in Breast Tumors and Correlates with Bim Expression

**Figure 1:**
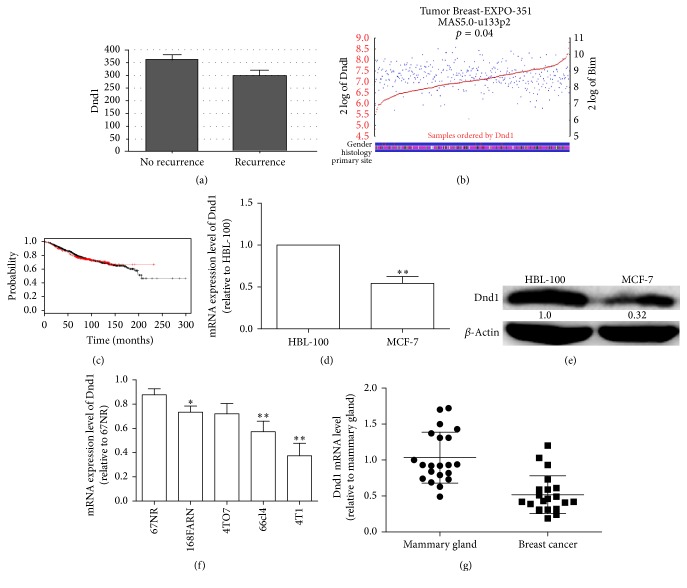
Dnd1 expression is correlated with Bim expression in breast cancer tissues. (a) After annotating the gene microarrays according to the TCGA microarray subtypes, we identified the expression levels of Dnd1 which were lower in recurrence groups than in no-recurrence groups. (b) The expression of Dnd1 and Bim in breast cancer tissues includes positive correlation (*p* = 0.04). (c) Correlation between the levels of Dnd1 and the survival of breast cancer patients. When the expression of Dnd1 was considered, patients in the Dnd1 low-expression group had a significantly shorter survival time (*p* = 0.007). (d and e) The Dnd1 expression levels were determined in normal and breast cancer cells by qRT-PCR and western blot. The expression levels of Dnd1 are higher in normal mammary gland epithelial cells than in breast cancer cells. (f) qRT-PCR analysis of Dnd1 mRNA with whole RNA extracted from mouse breast tumor cell lines with different metastatic potential. (g) Dnd1 mRNA level is decreased in human breast tumor samples. Twenty-one pairs of breast tumors with adjacent mammary gland epithelial tissues were analyzed by qRT-PCR. Values are mean ± SD. ^*∗∗*^ *p* < 0.01 (*n* = 3, one-way ANOVA).

mRNA microarrays were annotated by looking into the mRNA-based subtypes of breast cancer according to the TCGA. The mRNA expression was analyzed in the mRNA microarrays, and we found that Dnd1 expression level was downregulated in the recurrence subtypes compared with the no-recurrence subtypes (Figure 1(a)). Furthermore, the expression levels of Dnd1 or Bim are positively correlated (*p* = 0.04) (Figure 1(b)). The KM Plotter tool was employed to assess whether Dnd1 mRNA level correlated with the survival of breast cancer patients. The Dnd1 low-expression patients had significantly shorter overall survival than those of Dnd1 high-expression group (*p* = 0.014) (Figure 1(c)). Further qRT-PCR and western blot results exhibited lower Dnd1 expression levels in MCF-7 cells than in normal mammary gland epithelial cells HBL-100 (Figures 1(d) and 1(e)). And to examine whether Dnd1 levels were associated with tumor aggressive character, Dnd1 mRNA level was determined in five cell lines (67NR, 168FARN, 4TO7, 66cl4, and 4T1) with unique tumorigenic feature and increased metastatic capability in order [21]. As shown in Figure 1(f), Dnd1 exhibited the least level in 4T1 cells which is the most tumorigenic cells than in other cell lines. Finally, the Dnd1 level was further analyzed in cDNA samples from 21 pairs of breast tumors and their neighboring mammary normal epithelial tissues. An decreased mRNA level of Dnd1 was observed in breast cancer tissues with twofold lower than adjacent normal breast tissues (Figure 1(g)). These data suggest that Dnd1 could be a tumor suppressor in breast cancer and correlated with Bim.

### 3.2. Dnd1 Stabilizes Bim mRNA and Increases Apoptosis in MCF-7 Cells

**Figure 2:**
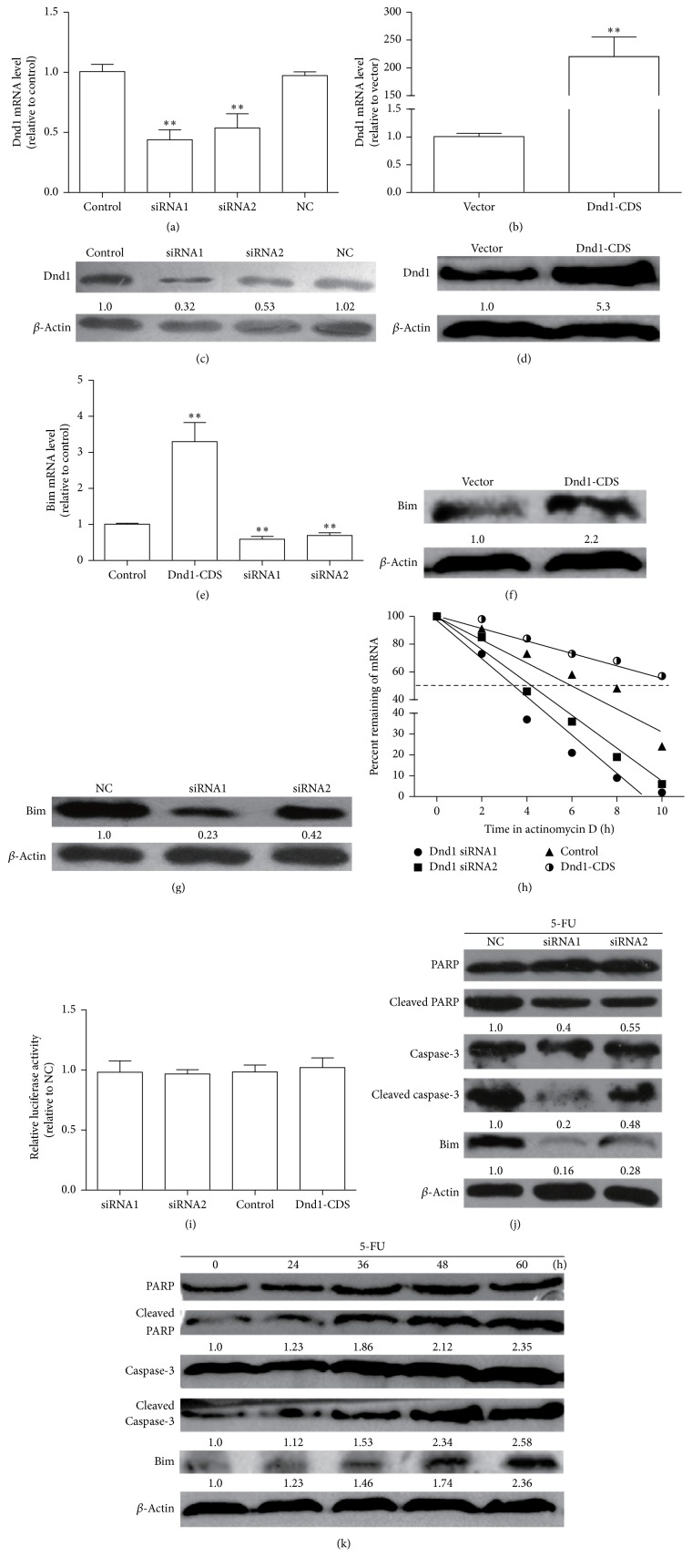
Dnd1 stabilizes Bim mRNA and increases apoptosis in MCF-7 cells. (a and b) The knockdown efficiency of Dnd1 siRNAs was analyzed by qRT-PCR and western blot normalized to *β*-actin in MCF-7 cells. (c and d) The transfection efficiency of Dnd1-CDS was analyzed by qRT-PCR and western blot in MCF-7 cells. (e) Forty-eight hours after transfection with 10 nM NC or Dnd1 siRNA, the amounts of Bim and GAPDH mRNA were determined by qRT-PCR. (f) Forty-eight hours after transfection with Dnd1-CDS, the Bim protein level was detected by western blot. (g) After treatment of MCF-7 cells with 10 nM NC or Dnd1 siRNA for 48 h, level of Bim was measured by western blotting. *β*-Actin was used as a loading control. (h) After a 48 h transfection of NC or Dnd1 siRNA, MCF-7 cells were treated with actinomycin D (2.5 *μ*g/mL) for the indicated times. Bim mRNA levels were measured by qRT-PCR and the percentage of mRNA that remained was plotted. (i) Dnd1 knockdown did not affect the promoter activity of Bim. After treatment with 10 nM NC or Dnd1 siRNA for 24 h, MCF-7 cells were transiently transfected with the luciferase plasmids containing the 5′-flank of the human Bim gene for 24 h. Luciferase activities in these cells were measured using the Dual-Luciferase Reporter Assay System (Promega, Madison, WI). (j) Whole-cell lysates were prepared from MCF-7 cells that had been treated with 10 nM NC or Dnd1 siRNA for 48 h. The levels of unprocessed or cleaved caspase-3 and PARP were measured by western blot. (k) Whole-cell lysates were prepared from MCF-7 cells that had been transfected with Dnd1-CDS for 48 h. The levels of unprocessed or cleaved caspase-3 and PARP were measured by western blot. *β*-Actin was used as a loading control. Values are mean ± SD. ^*∗∗*^ *p* < 0.01 (*n* = 3, one-way ANOVA).

As Dnd1 was a conserved RBP which could bind to the AU-rich sites in mRNA 3′UTRs [14], we hypothesized that Dnd1 could also bind to Bim-3′UTR, thus enhancing the stability of Bim mRNA and promoting the apoptosis of breast cancer cells. Firstly, we examined whether the stability of Bim mRNA was affected by Dnd1 knockdown. Dnd1-CDS and its siRNAs were transfected into MCF-7 cells for 48 h and remarkably upregulated or downregulated the mRNA and protein levels of Dnd1 (Figures 2(a)–2(d)). Meanwhile, the expression levels of Bim were increased in Dnd1-CDS-transfected cells and decreased in Dnd1 siRNA-transfected cells (Figures 2(e)–2(g)). In addition, Dnd1 was overexpressed or knockdown and then blocked* de novo* synthesis with actinomycin D; the decay rate of Bim mRNA in Dnd1 siRNA-transfected cells was faster than that of NC-transfected cells (*t*_1/2_ = 3.2 ± 0.3 h and *t*_1/2_ = 4.1 ± 0.3 versus *t*_1/2_ = 6.0 ± 0.4 h), whereas it is slower in Dnd1-CDS-treated group (Figure 2(h)). Notably, the luciferase reporter assay showed that Bim promoter transcriptional activity was not affected by Dnd1 ectopic expression (Figure 2(i)), demonstrating that Dnd1 could not alter the transcriptional activity of Bim. In addition, MCF-7 cells were used to examine whether Dnd1 knockdown could actually decrease the susceptibility to apoptotic cell death via decreasing the expression of Bim. Transfection of the Dnd1 siRNA into 5-fluorouracil- (5-FU-) treated MCF-7 cells prohibited the cleavage of poly (ADP-ribose) polymerase (PARP) and activation of caspase 3 (Figure 2(j)). Finally, to determine whether Dnd1 induced cell death by apoptosis, PARP and caspase-3 expression were further measured in cells transfected with Dnd1-CDS. As shown in Figure 2(k), Dnd1 caused cleavages of caspase-3 and PARP1 in a time-dependent manner. Overall, our results indicate that Dnd1 may modulate the proapoptotic properties of Bim and thus promotes the apoptosis in breast cancer cells.

### 3.3. Dnd1 Binds to Bim-3′UTR

**Figure 3:**
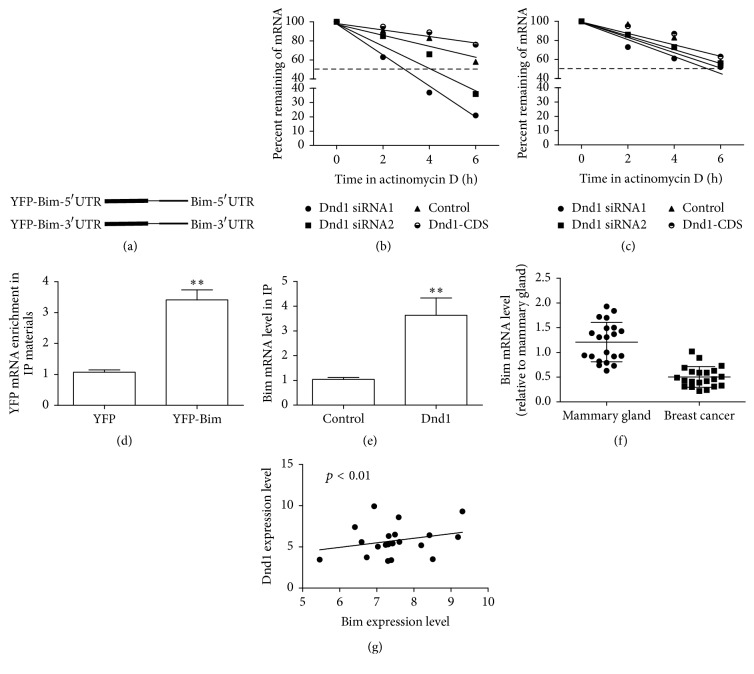
Dnd1 regulates Bim expression through Bim-3′UTR. (a) Constructs were prepared to express chimeric RNAs spanning the YFP with Bim-3′UTR or Bim-5′UTR as described in Section 2. (b and c) After MCF-7 cells were transfected with NC or Dnd1 siRNA for 24 h, the YFP-Bim-3′UTR (b) or YFP-Bim-5′UTR (c) vector was cotransfected for 24 h. These cells were then treated with actinomycin D (2.5 *μ*g/mL) for the indicated times. YFP mRNA levels were measured by qRT-PCR and the percentage of YFP mRNA that remained was plotted. (d) After a 24-hour transfection with the YFP or YFP-Bim construct, the amounts of Dnd1 binding to the chimeric RNAs were analyzed by RIP assay followed by measurement of YFP mRNA levels by qRT-PCR. (e) qRT-PCR was used to measure the abundance of Bim mRNA present in the Dnd1-IP materials after the RIP assay was measured by qRT-PCR. (f) The mRNA level of Bim was examined in twenty-one pairs of breast tumors with adjacent mammary gland epithelial tissues via qRT-PCR analyses. (g) Correlation between the levels of Dnd1 and Bim. Values are mean ± SD. ^*∗∗*^ *p* < 0.01 (*n* = 3, one-way ANOVA).

To further validate the association of Dnd1 with Bim mRNA, an YFP reporter construct of a chimeric RNA was constructed with bridging the YFP protein and Bim-5′UTR or 3′UTR (YFP-Bim-5′UTR; YFP-Bim-3′UTR; Figure 3(a)). MCF-7 cells were cotransfected with YFP-Bim-3′UTR and YFP-Bim-5′UTR plus Dnd1-CDS or Dnd1 siRNA. The degradation rate of YFP mRNA was detected with actinomycin D treatment via qRT-PCR. As shown in Figure 3(b), the mRNA half-life period of chimeric YFP-Bim-3′UTR was increased by Dnd1 overexpression but shortened by Dnd1 knockdown, whereas the decay rate of YFP-Bim-5′UTR did not change (Figure 3(c)). In addition, to determine whether Dnd1 bound to Bim-3′UTR, Dnd1 expression was induced in MCF-7 cells, and then Dnd1-binding complex with Dnd1 antibody was pulled down, followed by examining the bound mRNAs by qRT-PCR. As shown in Figures 3(d) and 3(e), RIP analyses indicated that the association between Dnd1 and the chimeric RNA containing Bim-3′UTR (Figure 3(d)) or Bim (Figure 3(e)) was more specially than control. Most importantly, qRT-PCR assays were performed to detect the tissue distribution of Dnd1 and Bim transcripts in normalized pooled cDNA samples from 21 pairs of breast tumors with neighboring mammary normal epithelial tissues. An decreased expression level of Bim mRNA was observed in breast tumor tissues over normal tissues (Figure 3(f)), and its expression was correlated with Dnd1 expression positively (Figure 3(g)). Overall, these results indicate that Dnd1 stabilizes Bim mRNA via interacting with Bim-3′UTR.

### 3.4. Dnd1 and miR-221 Competitively Regulate Degradation of Bim mRNA

**Figure 4:**
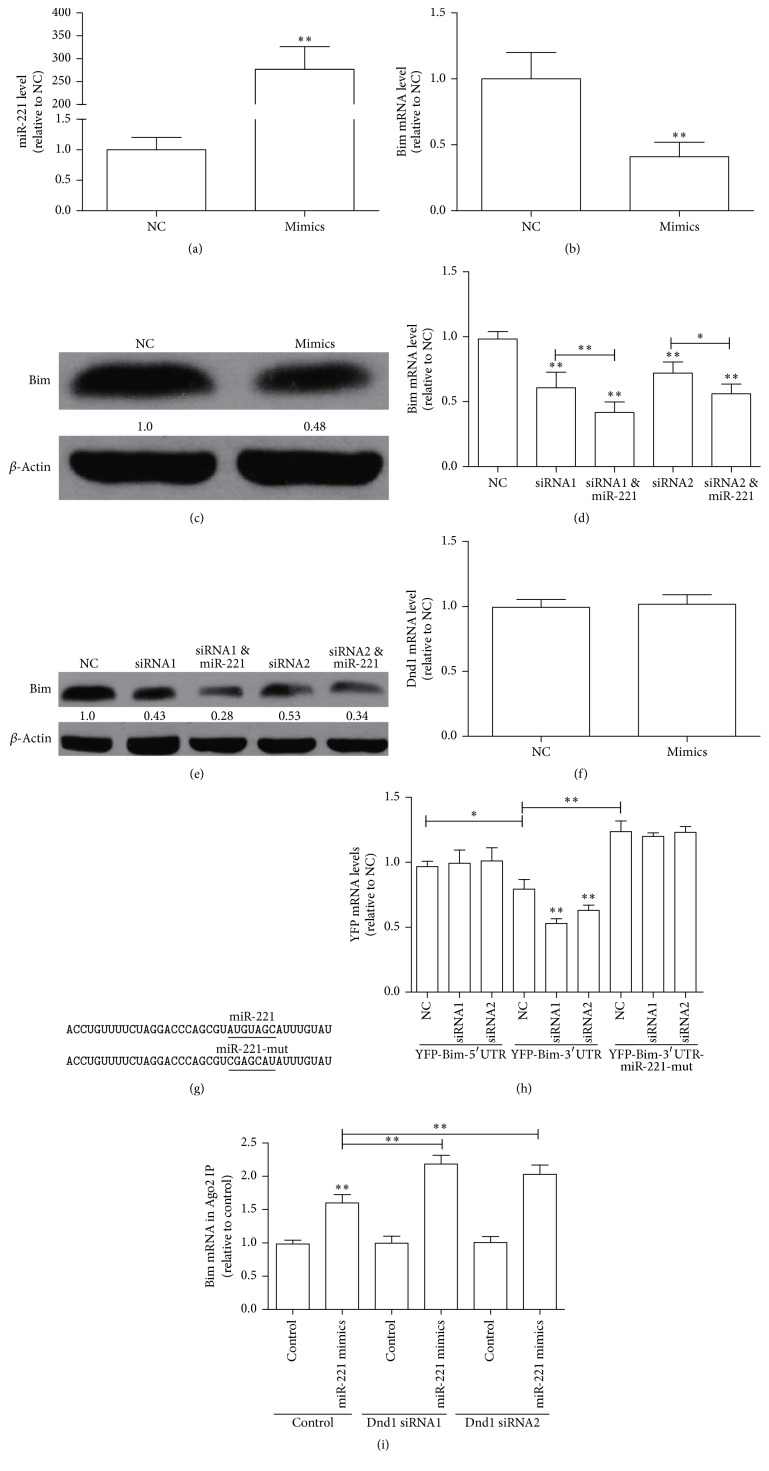
Coregulation of Bim expression by Dnd1 and miR-221. (a and b) MCF-7 cells were transfected with NC or miR-221 mimics for 48 h. The levels of miR-221 and Bim mRNA were measured by qRT-PCR. (c) MCF-7 cells were treated with miR-221 mimics or NC for 48 h. The protein level of Bim was detected by western blot. (d and e) MCF-7 cells cotransfected with NC or Dnd1 siRNA and miR-221 mimics for 48 h, the levels of BCL2 mRNA, and protein were measured by qPCR and western blot. (f) The mRNA level of Dnd1 in MCF-7 cells transfected with miR-221 mimics was examined by qPCR. (g) YFP reporter plasmids with Bim-3′UTR containing the intact miR-221-binding site (YFP-Bim-3′UTR) and a mutated miR-221 site (YFP- Bim-3′UTR) were generated. The mutated sequences are underlined. (h) After MCF-7 cells were cotransfected with NC or Dnd1 siRNA and each construct, then YFP mRNA levels were measured by qRT-PCR. (i) MCF-7 cells were treated as in (a and d). RIP assay was performed using an anti-Ago2 antibody or control IgG and lysates from cells transfected with the indicated oligonucleotides, and then Bim mRNA levels were measured by qRT-PCR. Values are mean ± SD. ^*∗∗*^ *p* < 0.01 (*n* = 3, one-way ANOVA).

As Bim had been proved to target mRNA of miR-221, the competitive effect of miR-221 and Dnd1 on the expression of Bim was further confirmed. The effects of Dnd1 siRNAs in MCF-7 cells overexpressing miR-221 were examined. As shown in Figure 4(a), MCF-7 cells transfected with miR-221 mimics could significantly increase the level of miR-221, and upregulation of miR-221 level significantly decreased Bim mRNA and protein levels (Figures 4(b) and 4(c)), which is consistent with the previous study that miR-221 can target Bim in breast cancer [17]. And Dnd1 downregulation in miR-221 mimics-transfected cells accelerated the miR-221-mediated degradation of Bim mRNA and thus decreased Bim protein level (Figures 4(d) and 4(e)). We also confirmed that Dnd1 mRNA level was not affected by upregulating or downregulating miR-221 level (Figure 4(f)). To accurately examine the interplay between miR-221 and Dnd1 on Bim-3′UTR, we constructed reporter plasmid that expressed chimeric RNAs including the sequences for Bim-3′UTR harboring mutated binding site for miR-221 and YFP (YFP-Bim-miR-221mut; Figure 4(g)). As shown in Figure 4(h), in cells transfected with NC, the quantity of YFP mRNA generated by the YFP-Bim-3′UTR vector was significantly less than the amount produced by the control YFP-Bim-5′UTR vector, which could be due to the miR-221 regulation and the fact that Dnd1 knockdown additionally accelerated the reduction of the mRNA level of YFP depending on Bim-3′UTR. On the contrary, the levels of YFP mRNA were reversed and the reduction was erased in cells expressing YFP-Bim-221mut, and knockdown Dnd1 did not affect the YFP mRNA level. Additionally, upregulation of miR-221 facilitated the association between Ago2 and Bim mRNA, which was further promoted by knockdown of Dnd1 (Figure 4(i)). Our results indicate that the binding sites of miR-221 and Dnd1 overlap, thus competitively regulating the degradation of Bim mRNA.

## 4. Discussion

Although the expression of Dnd1 is necessary for germ-cell development, little is known about its function in other physiological conditions, like breast cancer development. Here, the Dnd1 mRNA level was investigated in breast cancer tissues or cells and normal tissues or cells via qRT-PCR analysis. The results showed that Dnd1 expression level was lower in breast cancer cells or tissues and aberrant expression of the Dnd1 correlated with prognosis of patients with breast cancer. In addition, Dnd1 expression level was positively associated with the expression of Bim. And our further studies showed that knockdown of Dnd1 can lead to the decreased stability and expression of Bim but not affect the promoter activity of Bim. Finally, we provided evidences that Dnd1 protected the expression of Bim from repression of miR-221 by competitively binding to the of Bim, thus promoting the apoptosis of breast cancer. Our results pinpoint the mechanism by which Dnd1 exerts its function in breast cancer apoptosis. Binding of Dnd1 to Bim-3′UTR prohibits the interaction between miR-221 and Bim.

The mRNA stabilization is regulated by RBPs and miRNAs posttranscriptionally. RBPs modulate mRNA translation and stability by interacting mostly with the 3′UTR [22]. miRNAs frequently bind to “seed matches” in the 3′UTRs of target mRNAs resulting in translational inhibition or degradation of protein-coding transcripts [23]. Amount of evidence shows that RBPs posttranscriptionally regulate mRNA levels via the joint influence of miRNAs [24]. Such that miRNA processing is regulated by ADAR1 in a catalytically independent manner, which is required for differentiation and neural induction [25]. miR-21 is sequestered by HuR to prevent translation repression of proinflammatory tumor suppressor gene programmed cell death 4 in breast cancer cells [26]. Lin28A could enhance chemosensitivity of colon cancer cells to 5-FU by promoting apoptosis in a let-7 independent manner [27]. And MCPIP1 can selectively destabilize transcripts associated with an antiapoptotic gene expression program in breast cancer cells that can elicit complete tumor regression [28]. In contrast, let-7-mediated RISC could also be recruited to the 3′UTR of Myc by HuR and thus downregulates c-Myc expression [29]. Therefore, when the binding sites for RBPs overlap with or close to miRNA-binding sites, RBPs could either cooperate or compete with miRNAs via their physical interactions. Here, luciferase reporter assays showed that the binding sites for Dnd1 in Bim overlap with the miR-221 binding sites; therefore Dnd1 could compete with miR-221 in Bim-3′UTR.

Dnd1 plays a wide role in regulating gene expression and miRNA processing. It must be noted that, in addition to the competitive interactions between miR-221 and Dnd1, other potential mechanisms may be involved in the regulation for Bim expression. Nevertheless, the competitive binding between miR-221 and Dnd1 could give novel views into the modulation of Bim level, thus promoting the apoptosis in breast cancer cells. Based on the positive correlation between Dnd1 and Bim in breast cancer tissues, increased Dnd1 expression levels could act in concert with Bim or other tumor suppressors to promote breast cancer cell apoptosis, which indicates that Dnd1 possesses a tumor-suppressive role in breast cancer development. However, based on the fact that the functions of RBPs are different in various tumors, further animal studies should be performed to examine our conclusions, and it would be interesting to see whether this study could be extended to other tumors; thus then the results may have clinical applications.

In the present study, both miR-221 and Dnd1 posttranscriptionally regulate their target, Bim mRNA. Knockdown Dnd1 in breast cancer cells promotes Bim mRNA decay by competitive inhibiting the combination of miR-221 with Bim-3′UTR. Hence, knockdown of Dnd1 decreases the apoptosis of breast cancer cells and defining Dnd1 as one of the key players that regulate breast cancer development provides the possibility of controlling the apoptosis of breast cancer cells by modulating the expression of the Dnd1 protein.
